# Life satisfaction among Spanish children and adolescents participating in Physical Education

**DOI:** 10.3389/fpubh.2024.1370118

**Published:** 2024-05-22

**Authors:** Santiago Gómez-Paniagua, Antonio Castillo-Paredes, Carmen Galán-Arroyo, Jorge Rojo-Ramos

**Affiliations:** ^1^Faculty of Sport Sciences, BioẼrgon Research Group, University of Extremadura, Cáceres, Spain; ^2^Grupo AFySE, Investigación en Actividad Física y Salud Escolar, Escuela de Pedagogía en Educación Física, Facultad de Educación, Universidad de Las Américas, Santiago, Chile; ^3^Faculty of Sport Science, Physical and Health Literacy and Health-Related Quality of Life (PHYQoL), University of Extremadura, Cáceres, Spain

**Keywords:** life satisfaction, adolescent, children, Physical Education, students, school

## Abstract

Life satisfaction has been determined as a cognitive indicator of subjective wellbeing, a term that acquires vital relevance during adolescence as a protective factor against numerous psychological, mental and social disorders. Therefore, the objectives of this study are: (1) to evaluate differences in life satisfaction as a function of gender and school environment in Spanish children and adolescents; and (2) examine the possible associations between life satisfaction and age and/or body mass index (BMI) of the student body. For this purpose, a cross-sectional study was carried out with 723 students (aged 6 to 18 years) in which the “Satisfaction with life Scale” was applied, consisting of 5 items that measure self-perception of life satisfaction. Nonparametric statistics (Mann–Whitney *U* test) were used to explore differences in scores according to sex and school environment, in addition to Spearman’s Rho test to identify associations between scale scores and students’ age and BMI. Significant differences were obtained in terms of sex in favor of the male gender, and between the two environments of the centers in favor of the rural ones. In addition, the two variables explored (age and BMI) showed significant inverse associations with life satisfaction levels. Therefore, educational interventions and policies must take this information into account to design and develop actions aimed at improving this cognitive factor.

## 1 Introduction

Over time in scientific literature, three concepts have been used interchangeably to define an individual’s overall assessment of their life: happiness, life satisfaction (LS) and subjective well-being (SWB) (1). In this sense, Diener (2) pointed out that LS, as a mental indicator of SBW, can be understood as a cognitive process and judgment of one’s own satisfaction with life experience (3), or, in the words of Núñez (4), LS can be framed as a cognitive judgment about the quality of one’s own life in which the criteria for judgment are specific to each individual. Vennhoven (5) also made his contribution around the concept of LS, defining this construct as the degree to which the person positively evaluates the overall quality of their as a whole, being influenced by 4 different dimensions: overall LS, satisfaction with housing, satisfaction with finances, and satisfaction with social contacts. Conversely, a person’s sense of well-being is characterized by both feeling good and performing well; it also includes achieving one’s potential, taking charge of one’s life, having a sense of purpose, and being in happy and contented relationships (6). Within this description, it is important to highlight the multidimensional model of SWB developed by Diener et al. (7) and formed by four components: (1) global LS; (2) positive affect; (3) negative affect; and (4) satisfaction with specific domains of life.

In this context, different studies have pointed out several changes in the factors that determine well-being, therefore, the levels of LS have been characterized throughout the life cycle of different populations (8, 9). In particular, LS ratings show a U-shaped pattern in most of the research, with a reduction in LS during adolescence and early-middle adulthood, increasing drastically in older age (10). This U-shape has been defined as a paradox by numerous research teams, as some conditioning factors (such as monetary income) improve during the age range in which a worse level of LS is identified, while other indicators (such as general health) experience lower levels when the LS trend reverses at the end of the life cycle (11).

Adolescence has been identified as a stage of mental health risk in the life cycle (12), mainly due to the numerous physiological and psychological changes, in addition to the frequent disruptions in the social environment. Blakemore (13) defines it as the period of life between puberty and adult independence, which requires both individual development and cultural norms. During this particular stage, biological, psychological, cognitive and social changes may affect the LS assessment process, and self-concept may be considered an indicator of how adolescents cope with these changes in relation to LS (14). However, to the best of the authors’ knowledge, there is no scientific evidence that evaluates LS in early adolescence and how it evolves during the later years of early adulthood, as there are only longitudinal studies focusing on periods within the adolescent stage (15) or cross-sectional research focused on adolescents (16). The findings imply that the apparent decrease in SWB around the middle of adolescence may, in fact, start earlier and last longer throughout adolescence (15, 16). However, because most research have not yet looked at big age spans in adolescence or the transition between adolescence and adulthood, it is still unclear when exactly significant losses in LS begin. Following this line of thought, the school environment seems to have a great influence on the SL of children and adolescents (17). Academic results seem to be related to higher LS, with the practice of physical activity (PA) being the most important mediator for improving the mental health of students (18). Consequently, the Physical Education (PE) classroom becomes a fundamental context, since those students who show greater satisfaction with the subject experience greater intrinsic motivation (19), thus improving their academic results as well as their PA levels (20).

Similarly, numerous factors have been identified as relevant when analyzing the SL of children and adolescents. In this regard, differences have been found in terms of gender when analyzing this variable, although the results of the different studies differed. While several of them showed higher LS levels in the male gender (21), other studies, on the other hand, report no real differences in LS levels when sex is introduced as a variable for analysis (22). Likewise, the environment in which individuals reside plays a crucial role in their LS levels, although it is not a subject of widespread study among the scientific community. However, over the last few years, this topic has been gaining increasing interest and has given rise to a body of literature with diverse findings in various contexts (23). For example, Swami & Todd compared LS levels in Malaysian adults residing in rural and urban areas, with those individuals from rural environments exhibiting higher levels of LS (24). This trend has also been observed in migrants from rural to urban areas, who expressed a lower LS a few months after their relocation (25). Despite this, there is little literature that replicates these types of studies in adolescents and children (26, 27). On the other hand, overweight in children and adolescents is related to the development of a wide range of different pathologies, such as cardiovascular diseases or sleep problems (28). In addition, the consequences and complications of these diseases have a negative impact on the SWB of children and adolescents, reducing their LS (29, 30). In addition, psychological problems such as anxiety and depression can be considered as another factor to take into account in terms of reduced SWB (31), as well as the social problems they may suffer because of this status, since previous research has pointed to overweight schoolchildren as targets of behaviors such as bullying or marginalization (32).

Considering the enormous positive impact that LS has on the psychosocial development of children and adolescents, such as combating mental problems caused by stress or favoring situations of exploration of their environment (33, 34); and since LS is a concept with a higher reliability and stability compared to mood states when assessing SWB (35), this study focuses on LS with life as a cognitive indicator of SWB. Therefore, the aim of this research is to evaluate the levels of LS of PE students in primary and secondary education (from 6 to 18 years old) in a region in southwestern Spain (Extremadura), analyzing the possible influences of gender and environment of residence. In the same way, we also intend to explore the possible associations between LS and the variables age and BMI in this population. In this sense, and after reviewing the most relevant scientific literature on the subject, it is hypothesized that male students and those residing in rural areas will show higher levels of LS compared to their female and urban peers. Likewise, negative associations between LS and student age and BMI are predicted throughout the age period studied.

## 2 Materials and methods

### 2.1 Participants

This research followed a descriptive cross-sectional design. The sample size was selected following the non-probabilistic sampling method based on convenience sampling (36), since the purpose was to collect as many students as possible. Of the total sample (N = 723), 50.62% were boys and 49.38% were girls, so it can be considered that the sample was balanced in terms of gender. Regarding the location of the center, 47.99% studied in rural schools and 52.01% in urban schools. Rural schools were considered to be those located in towns with less than 20,000 inhabitants and urban schools those with more than 20,000 inhabitants, following the criteria established by regional public organization (https://www.dip-caceres.es/). The mean age was 13.08 years (*SD* = 1.78) and the mean BMI was 22.70 (kilograms of weight divided by height in meters squared) (*SD* = 2.39).

In order to participate in the study, two inclusion criteria were established: (a) To have the informed consent of the parents/legal guardians; (b) To be a student of the subject of Physical Education in Extremadura public educational institutions of Primary (between 6 and 12 years of age) or Secondary-Baccalaureate (between 12 and 18 years of age) education. Table 1 shows the sociodemographic characterization of the sample.

**Table 1:** Sample characterization (*N* = 723).

Variable	Categories	*N*	%
Sex	Boy	366	50.6
	Girl	357	49.4
	Primary Education	267	36.9
	Secondary Education	441	61
	Baccalaureate	15	2.1
Center environment	Rural	347	48
	Urban	376	52

Variable	*M*	*SD*
Age	13.08	1.78
Weight	57.90	15.13
Height	1.57	0.14
BMI	22.70	2.39

N, number; %, percentage; SD, standard deviation; M, mean.

### 2.2 Procedure

Firstly, the contact details of the schools providing Primary Education (6–12 years), Compulsory Secondary Education (12–16 years) and/or Baccalaureate (16–18 years) in Extremadura were obtained from the directory of Extremadura public schools. This register was provided by the Department of Education and Employment of the Regional Government of Extremadura.

Next, to establish contact with the Physical Education teachers of the schools included in the list, an e-mail was sent to the schools containing information about the study: (1) objectives; (2) model of the instrument; and (3) informed consent to be signed by the parents/legal guardians. All teachers who wished to participate in the study had to respond to the e-mail to make an appointment with a member of the research team who would supervise the completion of the questionnaires, in addition to obtaining the informed consent of the parents/legal guardians.

Similarly, when the researcher went to the center to administer the questionnaire through Google Forms with Tablets, he first checked that all the students had parental informed consent. In addition, before answering the questions, they were read aloud by the researcher to ensure that there were no doubts about the items and the instrument. On the other hand, the reasons for deciding to complete the questionnaire online were organizational and economic, so that all the data were collected in the database, facilitating their processing, and no money was invested in replicating the instrument. The questionnaires were administered to the participating students, whose data were collected anonymously, during the first academic term of the year 2022. The students took an average of 10 min to complete the instrument. The study was conducted in accordance with the guidelines of the Declaration of Helsinki and was approved by the Ethics Committee of the EDUCA platform for excellence in educational research (approval code: 42022).

### 2.3 Instruments

First, sociodemographic data were collected through a questionnaire of own elaboration in which six questions were included to determine the characterization of the sample: sex, age, height, weight, grade, and location of the center.

On the other hand, the Spanish version of the “Satisfaction With Life Scale” (SWLS) (37) was applied in order to analyze the students’ self-perception of their LS. The SWLS aims to assess the learner’s overall judgment of their LS as a cognitive indicator of their SWB (3). This instrument is designed on a 5-point Likert-type response scale with the following values: (1) strongly disagree; (2) disagree; (3) indifferent; (4) agree; and (5) strongly agree; to offer the respondent a variety of possible answers. Since the five items are all oriented in a positive manner, adding the five answers will yield the scale’s total score. Due to the unifactorial structure of the instrument, it showed good psychometric properties in children and adolescents (Cronbach’s alpha = 0.84) (37).

### 2.4 Statistical analysis

Initially, the assumption of normality was tested using the Kolmogorov–Smirnov test to analyze the distribution of the data. In this case *p* < 0.05 was obtained, therefore it was confirmed that the assumption was not fulfilled and that the most appropriate tests were non-parametric.

The Mann Whitney U test was used to analyze the differences in the scores in each of the SWLS items and as well as in their total score as a function of the sex or demographic location of the participants. A significance value was set for *p* < 0.05. Hedges’ g was used to determine the effect size of sex or demographic location for each SWLS item. Values less than 0.20 indicate no effect, values between 0.21 and 0.49 indicate a small effect, values between 0.50 and 0.79 indicate a moderate effect, and finally, values greater than 0.80 indicate a strong effect (38).

To determine the degree of relationship between LS and age or BMI, Spearman’s Rho test was used. For the interpretation of this statistic, we took into account the ranges established by Mondragón Barrera (39) who determined that coefficients between 0.01 and 0.10 determined the existence of a low correlation, values between 0.11 and 0.50 implied a medium degree of correlation, from 0.51 to 0.75 a strong correlation, from 0.76 to 0.90 a high correlation and above 0.91 the correlation was perfect.

Finally, the Cronbach’s Alpha and McDonald’s Omega coefficients were used to evaluate the reliability of the psychometric scales based on their internal consistency. To interpret the values reported, those established by Nunnally and Bernstein (40) were chosen, which indicated that values below 0.70 would correspond to low reliability, values between 0.71 and 0.90 would correspond to satisfactory reliability, and values above 0.91 would correspond to appropiate reliability.

Apart from that, the data collected in the questionnaires were processed with the statistical analysis software Statical Package of Social Science (SPSS) version 23 for Media Access Control (MAC). Likewise, the information on the variables analyzed was organized in tables indicating the total number (N) and percentage (%) in the case of the sociodemographic data, and the mean (*M*) and standard deviation (*SD*) for the scores obtained in each item and in the overall SWLS.

## 3 Results

Table 2 shows the descriptive data (from the mean and standard deviation) and the differences for each of the items that make up the SWLS as a function of sex and center location.

**Table 2:** Scores and differences obtained according to sex and center location of the items of the SWLS.

Items	Sex				Environment			
	Boy	Girl			Rural	Urban		
	*M* (*SD*)	*M* (*SD*)	*p*	*g*	*M* (*SD*)	*M* (*SD*)	*p*	*g*
In most ways my life is close to my ideal	3.82 (1.06)	3.65 (0.97)	<0.01*	0.17	3.84 (1.00)	3.64 (1.03)	<0.01*	0.20
The conditions of my life are excellent	4.35 (0.84)	4.15 (0.96)	<0.01*	0.22	4.29 (0.85)	4.22 (0.95)	0.57	0.08
I am satisfied with my life	4.46 (0.90)	4.27 (0.92)	<0.01*	0.21	4.44 (0.85)	4.30 (0.97)	0.04*	0.15
So far I have gotten the important things I want in life	4.25 (0.95)	4.12 (0.94)	0.02*	0.14	4.31 (0.87)	4.07 (1.00)	<0.01*	0.26
If I could live my life over, I would change almost nothing	3.78 (1.27)	3.53 (1.23)	<0.01*	0.20	3.66 (1.25)	3.65 (1.27)	0.99	0.01
SWLSS	4.10 (0.61)	3.89 (0.54)	<0.01*	0.36	4.12 (0.50)	3.88 (0.63)	0.03*	0.42

*p* is significant < 0.05*. *M*, mean value; SD, standard deviation. Each score obtained is based on a Likert scale (1–5): 1 “Strongly disagree,” 2 “Disagree,” 3 “Indifferent,” 4 “Agree,” 5 “Strongly agree.”

If the sex variable is observed, we can see that in all the items the boys obtained a higher score than the girls, with statistical significance in all of them as well as in the overall score. As for effect size, all differences could be defined as small except for item 4 (*g* = 0.14) In addition, regarding the environment variable, it can be observed that students whose educational center was located in a rural area showed greater LS than those belonging to urban areas, with a significant difference in items one, three and four, as well as in the final score. However, only item 4 (*g* = 0.26) and the final scale score (*g* = 0.42) reached a small effect size.

Table 3 shows the correlation between the different items of the SWLS and the variables age and BMI, using Spearman’s Rho test for its analysis.

**Table 3:** Correlation between SWLSS items and age and/or BMI.

Items	Age *ρ (p)*	BMI *ρ (p)*
Item 1	−0.04 (0.25)	−0.04 (0.22)
Item 2	−0.14 (<0.01*)	−0.12 (<0.01*)
Item 3	−0.16 (<0.01*)	−0.09 (0.01*)
Item 4	−0.17 (<0.01*)	−0.09 (0.02*)
Item 5	−0.05 (0.17)	−0.05 (0.18)
SWLSS	−0.13 (<0.01*)	−0.10 (0.01*)

*p* is significant < 0.05*. Each score obtained is based on a Likert scale (1–5): 1 “Strongly disagree,” 2 “Disagree,” 3 “Indifferent,” 4 “Agree,” 5 “Strongly agree.”

Regarding the data obtained for the age variable, inverse, significant and medium associations were found with items 2, 3 and 4 and with the global questionnaire score. Likewise, BMI exhibited significant inverse correlations when associated with items 2, 3 and 4, in addition to the overall scale score. However, only item 2 was characterized by a medium correlation, while the correlation for the rest of the items was low.

Finally, the internal consistency of the instrument was calculated using Cronbach’s Alpha (α = 0.82) and McDonald’s Omega (ω = 0.81) statistics. These values can be considered satisfactory following the recommendations of Nunnally and Bernstein (40).

## 4 Discussion

The objective of this research was to describe the levels of LS, as a cognitive indicator of SWB, of children and adolescents in the region of Extremadura, including for the first time in a single study a wide age range. Similarly, and after reviewing the expert bibliography on the subject, the possible influences of both gender and the school environment on these levels were analyzed. Finally, the possible correlations between scale scores and participants’ age and BMI were explored.

Regarding the gender variable, statistically significant differences were found in all items and in the overall score of the questionnaire in favor of boys. These results are consistent with previous findings by other researchers, for example Goldbeck and coworkers (41) studied the various domains of SWB in German adolescents, extracting significantly higher LS levels in the male gender. In the Spanish context, Fraguela Vale et al. (42) conducted a study with more than a thousand post-compulsory education students (16 to 18 years old), belonging to both public and private schools, identifying higher levels of LS in male students. Similarly, Reina Flores et al. (43) obtained the same relationship in a sample of 2,400 adolescents aged 12 to 17 years in southern Spain. These differences may occur, as experts point out, to girls experiencing higher levels of social support but tending to disclose negative feelings more frequently than men in everyday life (14), so that LS levels are equalized over the years. However, Aznar et al. (44) found favorable differences in favor of females in a large sample of adolescents in compulsory secondary education. In addition, there is another group of studies in which these differences in LS could not be appreciated when the gender variable was introduced (45, 46), even in Spanish-speaking populations in South American countries (47). This issue has already been discussed by previous research (48), which pointed out that the differences found in LS were not due to gender, but to economic status. Similarly, some researchers noted that despite the disparity in social opportunities, our society has made great efforts to distribute resources equally between men and women (49). This may have contributed to men and women’s balanced perceptions of LS (50).

Also, the environment in which a person resides and studies seems to have a great influence on an individual’s LS. To the authors’ knowledge, there is a paucity of scientific literature analyzing differences in SL according to the environment of residence in the adolescent population. In the present study, differences were found in rural students’ favor in three of the five items that make up the scale, as well as in their overall score. These findings follow the line of research conducted by Marquez and Long (51), in which they evaluated the levels of LS in 15-year-old adolescents in 46 countries, finding that in general levels those belonging to rural communities showed better levels despite the decrease experienced during the last few years. Likewise, Abreu and collaborators (26) pointed out the existing differences in LS in 757 adolescents from the north of Brazil, being the schoolchildren from urban areas those with better levels of LS. In this context, research conducted in India that explored the LS of university students found similar results, justifying the worse scores of urban students due to a frenetic lifestyle and the accumulation of stress (23). On the contrary, Li et al. (52) tried to explore the determinants of LS and its differences according to the environment of residence in Chinese adults, pointing to urban communities as those more satisfied with their lives. Additionally, they claimed that ancestor worship, financial stress, depressive symptoms, and ease of access to healthcare were all strongly associated with LS. Additional research also found differences in cognitive aspects between rural and urban areas. For example, prevalence of depression is higher in rural than in urban adults (53); likewise, SWB is more common in rural than in urban chronic patients (54). However, another study conducted on about thirty-five thousand Chinese adults reported no difference in assessing the residence environment as a mediator of LS (55). This is consistent with research in Spanish schoolchildren, where equality in LS scores was observed between the two environments (47). However, international researches obtain the same conclusion, the heterogeneity of the results indicate that the relationship between environment and LS varies between countries and regions (51), although earlier studies (56, 57) found some commonalities in determinants of teenage LS across nations and different factors. Thus, future studies should investigate the potential causes of these trends in teenage SWB and mental health outcomes, taking into account regional and national variations.

Regarding age, this research found a significant inverse association in three of the five items of the questionnaire as well as in its overall score. This question has been advocated by much of the scientific research in the field of SL, which advocates that SL declines in late adolescence and early adulthood, a decline that continues into middle age (8, 58). This trend was also seen by Orben et al. (59), who analyzed the evolution of LS from 10 to 24 years of age in both German and UK populations, finding evident signs of a decrease in this cognitive indicator of SWB during adolescence. Similarly, Goldbeck et al. (41) found a significant negative association between age and perceived LS in a sample of 1,274 German adolescents aged 11–16 years. Also, Aymerich et al. (60) showed that this decreasing trend began at 11 years of age when analyzing 600 Spanish adolescents, emphasizing the importance of psychological/affective care in the pre-adolescent and adolescent stages of life. Within this context, the possible causes underlying this decline in LS include shifting assessments of LS questions and rising social, financial, professional, or familial demands (61). Therefore, it can be emphasized that the results of the present study coincide with those presented in several studies of cross-sectional design (62), as well as those of longitudinal design (63), carried out with samples of adolescents. Experts explain this pattern by speculating that either the drop in LS scores during adolescence is a result of specific processes or that living situations during this time are becoming worse, such as increasing social insecurity, autonomy, or uncertainty (64). Though mental health and LS are not the same thing, adolescents suffer from mental illnesses like anxiety and depression and other aspects of SWB declines (65).

BMI has also been incorporated into the study as an influential variable in the LS of the participants, finding a significant and inverse correlation in 3 of the 5 items of the questionnaire as well as in their final score. These results are in line with those found by Baile et al. (66) who evaluated the possible influence of BMI on LS in 1200 Spanish adolescents, reporting lower levels of LS as student BMI increased. However, the CASPIAN-III study (67) found no relationship between BMI and LS in more than 5,000 Iranian students aged 10–18 years. Similarly, Tabak et al. (68) observed that adolescents with obesity reported lower levels of LS compared to their healthy peers, however the findings pointed to self-perceived body image as a mediator of LS and not BMI. Therefore, the studies developed in the Spanish context differ from those developed internationally. This could be because, despite the interpretative logic surrounding health’s implications that low weight should harm a person’s overall development, low weight is seen as a positive value in Spanish society, generating recognition and reinforcement that can mitigate the detrimental effects on health and have a positive impact on the subjective variables of quality of life and LS (69).

### 4.1 Practical implications

This research has shown the relevance of gender and school environment on students’ LS levels. Therefore, all interventions and programs proposed in a school context should consider these findings in order to improve this cognitive component of SWB. In this sense, implementing tasks or goals that are difficult to achieve will not only produce frustration in the students, but may also mentally affect this population, which is at a critical stage for their personal development. Following this current of thought, the teaching-learning process should be based on achievable goals for the students, being adapted according to the characteristics of the group, involving all educational agents to foster a positive, harmonious and motivating development environment. Interventions should also pay special attention to other psychological factors such as body image, the development of social relationships, the acceptance of low weight as a positive element or general mental health, as they have an important influence on LS, and therefore, on SWB.

### 4.2 Limitations and futures lines of research

The present investigation has certain limitations. Due to its cross-sectional design, the results should be interpreted with caution since cause-effect relationships cannot be developed. Also, the participants belonged to the same region of southeastern Spain, so there are several sociocultural factors that could affect the scale responses. In addition, only quantitative methods were used, although qualitative methodology could generate relevant information regarding other mediators of SWB.

As for future lines of research, it is proposed to conduct a longitudinal study in populations of wide age ranges in order to identify environmental factors that may decrease LS levels in this population, as well as to determine those critical periods in which more attention should be paid to the students. It would also be interesting to extend this study to other Spanish communities, which would make it possible to detect possible differences and adapt educational interventions and policies to each context. Finally, it would be interesting to adapt the questionnaire so that educational agents could indicate what they believe to be the LS levels of their students, so that the differences between the results provided by students and teachers could be reduced by means of intervention programs.

## 5 Conclusion

Significant differences were found in terms of student gender in all the items that made up the LS measurement scale as well as in the final score of the same, being the male gender the one with the best scores. Students in rural settings generally showed higher LS compared to their peers in urban settings. Similarly, both BMI and age showed a significant and inverse association with LS in students from schools in the region of Extremadura (Spain). These findings show how students’ SL can be mediated by different variables both intrinsic to the learners and extrinsic to them. Therefore, interventions should be designed, adapted and developed in different settings with specific objectives, so that the learner perceives higher levels of SL after the achievement of the interventions.

## Data availability statement

The raw data supporting the conclusions of this article will be made available by the authors, without undue reservation.

## Ethics statement

The studies involving humans were approved by the study was conducted according to the guidelines of the Declaration of Helsinki, and approved by the Ethics Committee of the EDUCA platform for excellence in educational research (approval code: 42022). The studies were conducted in accordance with the local legislation and institutional requirements. Written informed consent for participation in this study was provided by the participants’ legal guardians/next of kin.

## Author contributions

SG-P: Conceptualization, Data curation, Investigation, Methodology, Project administration, Writing – original draft, Writing – review & editing. AC-P: Funding acquisition, Investigation, Resources, Supervision, Visualization, Writing – original draft, Writing – review & editing, Validation. CG-A: Funding acquisition, Investigation, Resources, Supervision, Visualization, Writing – original draft, Writing – review & editing. JR-R: Investigation, Methodology, Project administration, Resources, Software, Supervision, Visualization, Writing – original draft, Writing – review & editing.
